# Theory of Sensation

**Published:** 1811-08

**Authors:** T. Smith

**Affiliations:** Bristol


### Medical and Physical Journal.

VOL. XXVI.] AUGUST, 1811. [NO. 150.

Printed for R. PHILLIPS, by E. Hemsted, Great New Street, Fetter Lane, London.

*For the Medical and Physical Journal.*

### *Theory of Sensation.*

(Continued from Vol. XXV. p. 475.)

### SECOND GENERAL PHENOMENON.

WHEN action of the vital power is interrupted by *excess* of those matters which in a certain quantity are necessary to action, sensation is experienced at the part where the interruption is, the efforts to act continuing.

### I. EXCESS OF CALORIC.

That a certain quantity of caloric is necessary to successful vital action is well known. But the important fact to which I beg leave now to call the reader's most serious attention, that excess of caloric proves a cause of obstruction to the actions of the vital power, is perhaps less generally understood; for, if I mistake not, increased heat and increased action are commonly employed as nearly synonimous terms. There appears, however, to be a very important difference, when the excess of caloric is productive of sensation.

#### *Experiment 1st.*

My hand being at the temperature of 94°, and moist in the palm, I took a large wine glass, and inverting it on the palm, observed that in less than a minute it became universally dim on the inside, from the perspirable matter condensing on the sides of the glass: at this time I had no sensation of either heat or cold in the palm of my hand. I then held the glass before a fire till it became somewhat warmer than my hand, and wiping my palm quite dry, I inverted the glass upon it, and held my hand so near the fire as to occasion a distinct, and rather painful sensation of heat in every part of my palm inclosed by the glass. I continued this for upwards of a minute, but the glass remained quite free of dimness, nor could I perceive the least appearance of perspirable matter having collected on the palm. On withdrawing my hand from the fire the sensation of heat soon went entirely off; and in less than five seconds the whole palm was studded with large drops of perspirable matter, which I could distinctly see through the glass begin to emerge from the surface of the skin the moment the sense of heat began to abate.

*Experiment 2d.*

Having freed my palm of any moisture, I again inverted the glass upon it, and instantly immersed both in water raised to the temperature of 120°, taking care that none of the water should insinuate itself under the glass. Having borne a very painful sensation of heat (which was felt nearly as painful in the palm under the glass as in the rest of my hand) for the space of a minute; I caused some cold water to be dashed over the glass in order to condense any vapour that might be contained within it. But no dimness or moisture was found either in the glass or on the palm.

*Experiment 3d.*

I immersed my hand, with the glass inverted on the palm as before, in water at 110°. The sensation of heat soon began to diminish. I kept my hand in the water exactly one minute after the sensation was no longer felt. Cold water was then dashed over the glass as before. It instantly became quite dim on the inside from the condensation of the vapour, and on removing the glass, drops of perspirable matter were distinctly seen on the palm; although not the smallest drop of the water in which the hand was immersed could have got into the glass from the manner it was held on the palm. The temperature of the water at the end of this experiment was 104°, that of the hand 98°.

These experiments, which were frequently repeated with the same results, show clearly that excess of caloric obstructs the action of the vessels by which the perspirable matter is thrown out. They show, no less clearly, that a sensation of heat is inseparably connected with obstruction of action from that cause; for when the action of the vital power was allowed to recommence by the removal of that excess as in the first experiment, or came to be successfully performed proportioned to the excess as in the third experiment, the sensation instantly ceased. But it is not the exhalent actions only only which appear to be obstructed by excess of caloric: it seems to hinder the action of the circulating vessels equally with that of the exhalents, as is shewn by the tumefaction and redness of a part suffering under a painful sense of heat. Besides, I find by experiments, that when a living part is immersed in very hot water, the water cools much more rapidly after the sensation of heat begins to diminish, than during the time that it lasts: and much quicker than when an equal quantity of dead animal fibre is immersed in hot water under similar circumstances; a fact which appears to prove, that while the sensation of heat continues, the actions by which the caloric is either carried into the system by the circulation, or made latent by some peculiar process, are less than after the sensation begins to diminish, or is no longer felt.

Taking it for granted then that the true cause of the sensation in these experiments was the actions of the vital power being interrupted by excess of caloric, we shall now inquire how far that principle may assist us in explaining other phenomena both in health and diseases*.

It is well known that when the heat of the body is above the natural standard and the skin dry, the sensation of heat instantly diminishes, on the surface at least, when a sweat breaks out. Dr. Currie, who seems to have been fully aware of the importance of sensation connected with temperature†, ascribes that effect to the perspiration, which is, no doubt, in a great measure, true. The perspiration beginning to flow when the heat of the body is excessive, is a proof that the vascular actions interrupted by the excess of caloric, are more or less restored, which appears from the third experiment related above to be the true cause of the sensation becoming less; for I know by frequent observations, that when the temperature of the palm of my hand rises to 96°, or even 94°, without perspiration, a burning sensation is felt in it. Whereas, at the end of the

---

* If it be objected that these experiments are performed on too small a scale to warrant our drawing any important conclusions from them, I have only to observe, that if the results they lead to were confined to the solitary facts they contain, I should have thought them unworthy of detail, but as leading to a general principle, applicable in *every* case, they deserve regard. The laws of life operate as certainly in the extremities as in the centre of the system, in a small part of the body as in the whole; just as the force of gravity is as conspicuous in the fall of a pebble as in the revolution of a planet.

† The manner in which our sensations are affected by changes of temperature, is a subject of importance, as well as difficulty; for without a more precise knowledge of this, the action of temperature on life cannot be understood.—*Medical Reports*, vol. I. page 133.

 third third experiment, the temperature was 98°, and in other experiments I have found it as high as 101°, without the least distinguishable sensation of heat.

Another known fact of great practical importance, seems to admit of an easy explanation on this principle; viz. that when the temperature of the body is above the natural standard attended with a painful sense of heat, the abstraction of caloric, within certain limits, does not produce a sense of cold equal to that which the same degree of cold applied would occasion, when no previous sensation of heat is experienced. The reason of which appears to be, that as the sensation of heat proceeds from action being interrupted by *excess*, and the sensation of cold from action being prevented by *defect* of caloric, so the abstraction of the excess, instead of diminishing, really increases action; and therefore, agreeable to the principle, ought to make the sensation of heat less, without causing an equal sense of coldness.

Here a fact, apparently irreconcileable to this, or indeed to any other general principle, will, doubtless, occur to the minds of some of my readers; viz. that in a species of fever first noticed by Dr. Currie, although the heat of the body is greatly above the natural standard accompanied with a distressing sense of heat, yet chilliness is produced by the slightest application of cold. This circumstance is not peculiar to that case of fever; it is mentioned by Dr. Cullen as attending the commencement of other fevers: I have frequently observed it in the eruptive stage of scarlatina, and have even known it take place sometimes in health.

In inquiring into the cause of this interesting phenomenon, it appears to me that we can make no advances to a just explanation of it without understanding the possible effects of great changes of temperature on the solids and fluids even of the living body. The following question naturally occurs on this occasion: when the temperature applied is so much above or so much below the usual standard as to produce a longer or shorter interruption to the action of the vital power, do no changes in the condition of the fluids or solids of the part take place, which would have been prevented if the action of the vital power had not been interrupted? In these circumstances does the excess of caloric produce no chemical decomposition of the animal fibre, or of the fluids circulating in the vessels? and may not a powerful abstraction of caloric induce very essential changes in the state of the fluids which it is not in the power of the principle of life immediately, if at all to remedy?

That increased temperature does not produce the same effects on the living as the dead body, *at least when the*

*powers*

*powers of life are permitted to act successfully*, is a truth too obvious to escape notice or require demonstration. But it occurred to me that if the principal of sensation here laid down be true, changes ought to be effected in the living body exposed to the action of caloric sufficient to produce a strong sensation of heat, nearly similar to those which take place in dead animal fibre exposed to the same temperature ; but modified perhaps as the degree of sensation indicated the actions of the vital power to be more or less interrupted.

That this is really the case is rendered probable by even a superficial review of facts. In inflammation of the intestines the rapidity with which gangrene ensues, to the symptoms which denote very great or total obstruction of the actions of the vital power, is sufficiently known. When any of the extremities is exposed to a very intense degree of cold, so long as to produce a permanent obstruction of the vascular actions ; the sudden exposure of the extremities in these circumstances to a high temperature is succeeded by the most excruciating pain, and the part obstructed becomes rapidly gangrenous. My readers will, no doubt, be able to recollect many similar instances, in which the application of caloric seemed to produce or hasten disorganization, and I suspect, in all such cases it will be found by the state of the sensations, that the plastic or preserving powers of life are prevented from acting successfully.

But even when the sensation of heat is very slight, some degree of chemical decomposition of the animal fibre can, in some instances, be detected to have taken place. In order to exhibit the comparative effects on the fibre of caloric, when it does, and when it does not induce sensation, the following experiments are selected, chiefly for their simplicity and the ease with which they can be repeated.

### *Experiment 4th.*

At a time when the palm of my hand was without sensation and bedewed with perspirable matter, I inverted a wine glass upon it nearly full of water at the temperature of 90°. A slight sensation of warmth was felt at first, but this soon went off. I agitated it frequently, and at the end of a minute took off my hand and filled the glass with lime water, but no milkiness ensued although it stood several minutes. I varied this by collecting a considerable quantity of perspirable matter in a glass containing some lime water. But in no instance could I perceive the least marks of carbonic acid having been produced when I had no *sensation of heat* in my palm.

*Experiment*

### *Experiment 5th.*

Having put into the glass the same quantity of water as in the last experiment, at 120°, I inverted it on the palm of my hand which I had previously wiped dry. The sensation of heat was almost intolerable, but I bore it for one minute, agitating the water from time to time as before. The glass being now filled up with lime water, the mixture became somewhat cloudy at top, and at the end of three minutes was quite milky.

I made trials with water at 96°, and upwards, and it is curious that the production of carbonic acid seemed always proportioned to the sensation the water caused; the milder the sensation of heat the less carbonic acid being produced, and *vice versa.*

To some this experiment may appear a proof that the vital power did really act on producing the carbonic acid, but that the mode of action was different when the sensation was felt, and when the palm freely perspired without sensation. The following experiment appears unfavourable to that supposition.

### *Experiment 6th.*

I procured a piece of membrane of an animal lately killed, and having washed it and allowed it to lie for some time in water at 96°, I dried it well and put it over the mouth of a glass containing the same quantity of water at 120°, as in the fifth experiment. Having inverted the glass upon it, I allowed the membrane to suffer the action of the caloric for one minute, during which I agitated the water repeatedly. Filling now the glass with lime water, I observed the mixture immediately assumed a bluish cast, and after having stood three minutes it appeared even more milky than in the last experiment. Even when water at 90° was used the quantity of carbonic acid seemed very little less.

It will not be contended that the production of carbonic acid in this last experiment was owing to any but a chemical process, aided or excited by caloric. But since it was proved by the first three experiments that the vital actions are obstructed when a sensation of heat is felt; since no carbonic acid was produced when no sensation was felt, although what are incontestably actions of the vital power were going on, as in the fourth experiment; and since the more sensation was experienced, the nearer the quantity of carbonic acid produced in a given time, approached to that which is obtained from *dead* animal fibre; the conclusion appears irresistible that that the carbonic acid formed in the fifth experiment was owing to mere *chemical* actions of the fibre, aided or excited by the caloric.*

But if chemical changes, similar to those which take place in dead animal fibre, are effected in the *solids* of the living body by the agency of caloric, when the vital power is prevented from acting; it cannot be doubted that the *fluids* also must be more or less decomposed or changed in these circumstances. That this is the case seems to be in some measure confirmed by the peculiar changes the blood is known to undergo in typhus and inflammatory fever; in both of which the state of the sensations indicate, that the actions of the powers of life are very seriously interrupted, though the interruption is specifically different in each and may exist in different degrees.

These things being granted, the cause of the phenomenon that led to this discussion may be conceived to be, that, possibly, during the cold stage certain chemical changes are produced in the cutaneous vessels, by which the capacity of the fluids for caloric is greatly diminished, and consequently the fluids condensed and rendered less moveable. In consequence of the diminished capacity for caloric, the skin will be both more easily heated, and more easily cooled, than in the natural state; and in consequence of the obstructed state of the vessels the actions excited by excess and defect of caloric will be equally interrupted.†

Other facts might be adduced in support of the principle of sensation, as far as regards excess of caloric; but enough has already been said, I hope, to prove that the action of the *vital* power is interrupted when a sensation of heat is felt.

---

* The perspiration of carbonic acid by the skin (as a vital process I presume) has been affirmed by some philosophers, and denied by others. See Dr. Kellie on the Functions of the Skin, Edinburgh Medical and Surgical Journal, vol I. page 174. Though carbonic acid is not perspired by the palm of the hand, I do not assert that it may not be so by the skin of the axilla. At the same time I cannot but suspect, that the discordant results obtained in the experiments of these philosophers, might be, in a great measure, reconciled by attending to the *sensations*.

† I wish it to be understood, that no more stress is laid on this explanation than shall be justified by future observations or experiments. I have ventured the above to show that the phenomenon noticed by Dr. Currie, and others, *may* be explained on the principle of sensation. At the same time, till the existence of the state of the extreme vessels and fluids alleged to be present in these cases, is satisfactorily proved; it appears most prudent to suspend our judgment, and hold that phenomenon neither a proof nor exception to the principle.

This

This being established, the following inference will, I think, be found justly drawn from it; viz.

*That when the excess of Caloric is abstracted by means of cold applications, vital action is truly increased, provided always the abstraction be carried no farther than to remove the sensation of heat, and not to produce a painful sensation of cold.*

In gout the topical application of cold to the inflamed joint within the limits here mentioned (which are exactly the same as Dr. Kinglake has strongly and repeatedly insisted on*) is, I apprehend, attended with no danger of destroying or repelling the gouty action, whatever that be, but on the contrary permits the actions to be more successfully performed, by removing a powerful cause of obstruction. Whether the first effect of the morbid actions is considered to be *torpor*, which it is the intention of the increased evolution of caloric to remedy; or that the primary effect is a greater evolution of caloric, which, if I mistake not, is Dr. Kinglake's idea of the disease; still, in either case, the abstraction of that portion of the caloric which produces painful sensation, promises to be equally safe and efficacious. If the first be the proximate cause of gout, the painful sense of heat proves that so much caloric is accumulated in the part as to obstruct the actions of the vital power, therefore the removal of the redundant caloric, by restoring impeded vascular action, must promote the intention of nature, and allow it more speedily to overcome the morbid torpor. But if the increased evolution of caloric be both the cause and effect of the disease, or in other words, the disease itself, the application of cold under the guidance of the sensations, instead of diminishing the quantity evolved in a given time, does really, I strongly suspect, increase it. This may be understood by observing the effect of secreted fluids accumulating at the mouths of secreting vessels. In an abscess the quantity of pus secreted in a given time, will, *cæteris paribus*, be always greatest when the flow of matter is least resisted. If the urine were prevented from passing into the bladder by calculi in the ureters, and were thereby caused to accumulate in the kidneys, it is not possible so much urine could be secreted in a given time as when the ureters are pervious; for the urine pressing against the mouths of the secreting vessels, must greatly, if not entirely, prevent their action; the same is obvious of all other secretions. Applying this reasoning to the accumulation of caloric in gout, it follows that the applica-

* See Dissertation on Gout, &c. by Robert Kinglake, M. D. See also Additional Cases of Gout, by the same Author.

tion tion of cold in the manner directed by Dr. Kinglake, does really facilitate the morbid action, and consequently allow it to exhaust itself more speedily and with less derangement of the organization of the part.

Hence, I conceive, that topical refrigeration in gout, *so far as it removes the painful sense of heat*, must prove, not only safe, but highly beneficial.

But if the abstraction of caloric is carried so far as to induce a painful sensation of cold, and especially that state of the part, in which no sensation of cold is felt although it is below the temperature of health, I suspect that very different consequences might ensue; for although Dr. Kinglake seems to make light of any fears which may be entertained of repelling gout to the stomach or other important organs; still it must be acknowledged, that recorded facts strongly countenance the old opinion, and with these facts any knowledge we have of the effects which the excessive abstraction of caloric has upon the vital actions seems to correspond. I cannot therefore but suspect, that the metastasis of gout from the extremities to the stomach, brain, or other important organ, in consequence of the injudicious application of cold, is an evil really to be dreaded, and carefully to be guarded against.

But although too much refrigeration may have sometimes forced nature to abandon her post, this should not deter us from employing so much as by experience is known to quicken and facilitate her operations: which cold applications certainly do when they remove painful sensation and diminish the swelling of the part, these effects being direct consequences of increased action of the powers of life.

Quere. In cases where the cold applications prove disagreeable to the sensations, might not topical blood-letting, by unloading the overdistended vessels, promote the intention of cold applications, and render these more grateful to the feelings?

With regard to the application of cold in inflammatory affections of the viscera, might it not, especially in inflammation of the intestines and peritoneum, be sometimes employed as an useful auxiliary? What possible harm can result from abstracting that portion of caloric which produces a painful sense of heat? It certainly merits the most serious consideration of those to whose charge the lives of their fellow creatures are committed, what must be the destructive consequences to the organization, of caloric being allowed to accumulate in an important organ in such excess, as to greatly diminish, or totally prevent, the action of that power upon which the support of the organic fabric depends. That *chemical* actions destructive of the fluids or solids of the living body, cannot take place when the *vital power* is allowed to act successfully, is a position which, I hope, will be the more confirmed the more it is reflected on. That the occurrence of such chemical actions is to be dreaded in every case of painful sensation, attended with excess of heat, I strongly suspect, both from the experiments and observations above related, and from a review of other facts, which it is unnecessary to specify, because many such must undoubtedly occur to the mind of every medical observer: and therefore, in enteritis especially, I should think it desirable and adviseable to remove the *excess* of caloric, which, although not the first cause of the disease, does without doubt, in many instances, increase its ravages and hasten its fatal termination.

I meant to have given other examples of sensation caused by action being interrupted by *excess* of those matters which are necessary in a certain quantity to action; but I have dwelt so long on the subject of caloric, and that being the most important, I shall pass over the others for the present, and hasten to the next general phenomenon.

### THIRD GENERAL PHENOMENON.

When one action of the vital power interrupts another, sensation is felt at the part, the efforts to perform the obstructed action continuing.

### 1. Actions interrupted by the voluntary power.

Every muscle is supplied with innumerable small vessels, by the united actions of which, it is taken for granted, the muscular fibres are preserved in a condition proper for contraction and relaxation. In our last paper it was shown that the contraction of the voluntary muscles is, in general, not attended with sensation in the fibres acting; this, if I am not mistaken, will be found to be always the case, when the contraction interrupts none of the actions of the muscular vessels: for,

1st. When a muscle in a state of inflammation is stimulated by the voluntary power to contract, a very painful sensation is immediately felt in the muscle. This pain cannot arise from *increased* action, for the action of the voluntary power in this case is not greater than in other cases in which no sensation is produced: nor can the action of the muscular vessels be then increased when they are compressed by the contraction of the muscular fibres. The pain, therefore, obviously arises from the interruption the actions of the new and delicate vessels suffer from the mechanical pressure of the contracted fibres, because similar pain is caused by external external pressure although the muscle be in a state of relaxation.

2d. When a muscle is spasmodically contracted, the violent pressure which the small vessels, of which it is almost entirely composed, sustain during the excessive contraction, (under which the muscle feels as hard as a board) must totally interrupt the actions of these vessels; accordingly, excruciating pain is felt, which immediately abates when the pressure is removed. But, as may be expected, some soreness remains; because it is not to be supposed that the action of these vessels can be instantly performed in *perfection*, after such violent pressure, by which, undoubtedly, their elasticity and perfect organization is more or less impaired; and therefore must require some time to refit. Or granting that the organization is not impaired, it must require some time ere these vessels can be again supplied, as before, with fluids, without which it is utterly impossible their functions, although attempted, can be performed. I could wish this to be well understood, because I think it applies in every case of the same kind. In cramp of a muscle there is doubtless *increased action of the power which contracts* the muscle, but this increased action is not necessarily attended with sensation. It is not the power which produces contraction, but the power whose actions are prevented by the contraction of the muscular fibres that feels.

3d. Let a person keep the flexor muscles of his thigh in a constant state of contraction for a few minutes, he will soon feel a sensation of fatigue in them, incomparably sooner than he would do if these muscles were moderately contracted and relaxed alternately, as in walking. If the power of volition acting on muscular fibres impairs their elasticity and mobility; and if it be the part of the vessels of the muscle to restore these; it is self-evident that function cannot be performed so long as the muscle is contracted. Hence, it is plain, the elasticity, &c. of the muscular fibres, will not only be more impaired, but the action of the vessels which repair these, more obstructed for the time when a muscle is permanently contracted, than when it is alternately contracted and relaxed: because under relaxation the small vessels being permitted to act will upon the whole suffer little or no interruption of their function, at least, if the pressure during contraction is never so great as to impair their power of acting. Hence the great length of time a moderate and equal pace in walking is often continued without fatigue; and hence most people in performing a journey on foot, experience uneasy sensation in the soles of the feet, or in the joints, much sooner than in the muscles employed in walking.

### 2. By stimulated actions.

1. It cannot have escaped the notice of those who have examined into the sensations of patients during the operation of medicines, that they often bear no relation to the *actions* these medicines are expected to produce. Thus it must have been observed that a cathartic medicine has sometimes produced copious evacuations without either sickness, gripes, or other sensation at all proportioned to the increased action that must have taken place; nay, that in some cases the operation of the medicine has been effected without producing the smallest degree of either griping or sickness; while, in other cases, severe sickness or violent pain, or both, have been felt, neither attended nor followed by evacuations. These facts cannot, I am afraid, be reconciled, unless it be allowed that sensation and action are different and opposite conditions of the vital principle. If I have not been deceived in my observations, the following are the rules by which sensation or action is produced by an evacuant medicine. 1st. When contraction equal to the stimulus does not take place, *sickness* is felt. 2d. When contraction equal to the stimulus is effected, but the action of the muscular vessels obstructed by the contraction, simple *pain*, but no sickness is felt. 3d. When the stimulated action (without interrupting any other) is immediately performed equal to the stimulating cause, neither sickness nor pain is experienced. Whether or not these rules are correct, must be decided by future observations.

2. Effects similar to the above sometimes take place very distinctly on applying the galvanic stimulus to any part of the body. I have repeatedly observed contraction induced in a muscle at some distance from the part galvanized, although not the least sensation was perceived either in the part to which the stimulus was applied, or in the muscle contracting*. That sensation is experienced in the muscle when it contracts very strongly is not to be wondered at, in that case the action of the muscular vessels must be more or less obstructed by the mechanical pressure of the contracted fibres.

3. When the stomach is stimulated by any means to contract suddenly and expel its contents; if the contraction is

---

* Dr. Mongiardini made the same remark. "The contraction," he observes, "is not always in direct proportion to the pain felt by the patient. It is even not rare to induce the contraction without the sensation, and vice versa.—*Edin. Med. and Surg. Jour.* vol. iii. page 32.

performed performed easily, quickly, and in proportion to the stimulus, no sensation is induced but what may be referred to the mechanical pressure of the contents of the stomach on its internal coat. But if the contraction does not immediately take place as it ought, an increased flow of saliva, or contraction of the vessels of the skin, or sickness at stomach is felt, which immediately ceases when the stomach contracts; but if this contraction be strong, lasting, and unsuccessful, a violently painful sensation is felt in the stomach, (without sickness) which obviously arises from the interruption the pressure of the contracted fibres gives to the action of the vessels in the substance of the stomach; because although the pain abates when relaxation takes place, yet some degree of soreness commonly continues for some time, owing, I conceive, to the interrupted actions not being immediately fully restored.

In more violent cases of cramp of the stomach, is there not reason to suspect that sometimes a permanent obstruction of the actions of the compressed vessels remains even after the relaxation of the muscular fibres; and that violent unsuccessful efforts to restore these actions produce rupture of the vessels, and effusion of the fluids contained in them? How far such an obstruction proves a stimulus to the production of new vessels, or, in other words, to inflammatory action, deserves to be inquired into.

4. The pain that occurs in strangulated hernia, colic, and other cases of obstructed intestine, is so similar to that which is felt in the above instances, that it cannot but depend on the same cause, mechanical obstruction to the action of the vessels of the gut caused or increased by the contraction of the muscular fibres: remove the obstruction in these cases by which the progressive motion of the contents of the intestine is prevented, and the pain will be no longer felt, although the *action* of the gut continues really as great as before; provided inflammatory action has not come on.

When the action of the small vessels of the heart is prevented by spasmodic contraction of that organ, what symptoms attend? Did not the violent pain, with complete loss of pulse, which occurred in Mr. John Hunter's case, arise from that cause?* If so, I suspect the recovery from that attack was owing to the pain continuing till the obstruction was overcome†.

* See Mr. Home's account of Mr. Hunter's life prefixed to the Treatise on the Blood, &c. page 46.

† For the pain indicated, not only obstructed action, but that the efforts to act continued unimpaired; had these efforts ceased, the pain would have ceased, but the consequence must have been fatal.

5. It

5. It has often been remarked by very judicious and accurate observers that the seat of sensation is not always the seat of the disease which gave rise to it. In making this remark, they only stated a fact, and I believe had not the most distant intention to infer from it either that sensation could take place without a cause, or to discourage any attempts that might be made to discover the laws by which it is governed. Yet that remark has induced some to believe that the sensations are altogether fallacious and not to be trusted to; an opinion which, if it were strictly followed in practice (which it is not, I hope, even by those who urge it most) could not fail to produce the most pernicious consequences. The following are the principal facts which have led to that opinion. 1st. Pain of the head from disordered stomach. 2d. Of the shoulder in hepatitis. 3d. Of the arm in dropsy of the chest, or aneurism of a large vessel there; or from an extraneous body getting into the trachea. 4. In the chest, from flatulence in the intestinal canal. 5th. Cramps in the muscles of the thighs and legs in cholera and other violently painful affections of the contents of the abdomen. 6th. Pain in the groin and testis in nephritis. 7th. In the glans penis from stone at the neck of the bladder. And 8th. In the bowels, chest, or head, &c. from coldness of the lower extremities.

We can only judge of the nature and cause of these pains from observing the attending circumstances. I have known the headachs from disordered stomach cease immediately when pain of the stomach came on. The pain of the shoulder in hepatitis commonly alternates with that in the region of the liver. The pains in the chest from flatulence instantly cease when the action of the intestine returns: thus I have repeatedly known such pains go off when the gut contracted, which it was known to do, not from any sensation felt in it, but from the gurgling noise caused by the motion of the air within it. In cholera the cramps of the muscles of the lower extremities alternate with those in the bowels; the patient being sometimes seized with the one the instant the other leaves him. The nature of these, and of all the other pains, seems to be the same. In all of them the action or attempt to act, ceasing in the original part, an action takes place in some other part of the system which is attended with sensation or not, according as it interrupts or increases action there. Thus some persons, when they sit with their feet cold or damp, are seized with a catarrhal increase of mucous discharge in the nostrils, which produces no sensation but what is accounted for by the irritation of the thin mucus; others in the same circumstances feel sensation although the discharge of mucus is diminished. In others, again, damp feet produce duce spasmodic pains in the bowels; in others diarrhœa without pain: in others the same cause induces a fit of asthma; in others headach; in others inflammation of the throat. In all these cases this circumstance, as far as I have been able to trace, occurs in common. The sensation of cold ceases, or becomes less in the extremities (though the temperature is not increased) before the symptoms of the secondary affections begin; and if the sensation of cold in the extremities continues steady, the secondary disease does not take place although the circumstances are the same in every other respect. From all these circumstances we cannot but infer, that the painful sensation in all the above instances is produced by spasmodic actions, and that the spasm arises from the efforts to act in the original part ceasing and removing to some other part, with the effect of interrupting the usual action there; hence the pain; but that if instead of interrupting it increases action, no pain is induced, but only the effects which characterize increased action, viz. increased discharge of fluids.

### 3. By the mental actions.

There are few strong emotions of mind which do not give rise to action, either in the voluntary or involuntary muscles. Of the actions accompanying the different passions, some produce sensation, others none; and even the action succeeding to the same mental affection sometimes produces very distinct sensation, while, at other times, no sensation is induced, although the fibrous motion is equally visible. I shall instance only one mental affection at present; and if it shall appear that the sensation produced in it is truly a consequence of interrupted, or, in other words, spasmodic action, it will not, I hope, be judged an abuse of analogy to infer, that the sensation produced by other mental affections arises from the same cause.

Many persons in reading a sublime piece of poetry, or hearing a fine air in music, if they are much delighted with any particular passage in the poem, or cadence in the music, feel a sudden chilliness all over as if cold water were poured over them. I know one person in particular who, in reading Pope's Elegy to the Memory of an unfortunate Lady, was often seized with *cold shivering*, during which the papillæ of the skin, particularly in the arms and back, became elevated as in the cutis anserina. Nearly the same effect is produced in many by music which touches their fancy. But it is a remarkable circumstance that sometimes, instead of a sensation of coldness, the skin feels only as if it were strongly contracted, and at other times not the least sensation can be perceived, ceived, although the contraction of the skin is visible to the eye, and though the emotion of mind producing the motion in the skin is exactly the same. This difference shows decisively that the sensation must have depended on some other cause than mere action, otherwise it should have been the same in every case. The circumstances giving rise to these differences appear to be the following. When the heat of the skin is natural, the action succeeding to the mental emotion causes a sensation of coldness. When the heat of the surface is natural, but supported by artificial means, the sensation of contraction only is felt. When the skin is hot and dry no sensation of cold is felt, but the sense of heat diminishes, and a sweat breaks out.

Are we to conclude from these effects that there are actions by which caloric is made directly latent? I have been able to ascertain by the thermometer that in these cases the temperature of the skin experiences a considerable fall; in some instances it has sunk half a degree, in others two degrees as soon as the cutaneous motion became visible. But the effect of the mental emotion is so transient, that it is not possible to form a certain judgment how much caloric is carried off. If the existence of actions of the vital power by which caloric is made latent were ascertained on proper evidence, it would account for the sudden fall the temperature of the body sustains in the cold fit of an ague; it would also account for a fact I have observed in plunging into the cold bath, that the system seemed to lose more caloric than was gained by the water, although a standard from which to judge was attempted to be formed, by observing the power of dead animal fibre in communicating temperature to a proportional quantity of water. But experiments to determine this interesting question are necessarily complicated with so many circumstances that may prove a source of error, that I am not satisfied with any trials I have yet made to determine it, and it appears to me too important to be either entirely disregarded, or taken for granted without further investigation.

But in whatever way this question shall be determined, it cannot affect the following conclusion from the different effects of the same emotion of mind in the circumstances above mentioned, that the sensation of coldness, when it was produced, depended solely on the interruption which the action of the mental power occasioned to the other actions of the skin, by the abstraction of some portion of caloric which was necessary to successful action.

Upon the above mentioned effect of the emotion of *delight* occasioning a flow of sweat, when the heat of the skin is above the natural standard, might, perhaps, be explained the influence fluence ascribed by the ancients, and some of the moderns, to music in the cure of certain diseases. See Mead on Poisons, Essay 2d. I can only speak from experience of its effects in what has been termed nervous head-ach, attended with febrile heat of body, in which I have repeatedly known a strong, lasting, and agreeable emotion of mind, *by whatever cause excited*, act like a charm; it produced perspiration, and the head-ach vanished, not to return even after the mind was no longer occupied by the pleasing emotion.

T. SMITH.

*Bristol, June* 3, 1811.

